# Vitamin D Modulates the Response of Bronchial Epithelial Cells Exposed to Cigarette Smoke Extract

**DOI:** 10.3390/nu11092138

**Published:** 2019-09-06

**Authors:** Carolien Mathyssen, Jef Serré, Annelore Sacreas, Stephanie Everaerts, Karen Maes, Stijn Verleden, Lieve Verlinden, Annemieke Verstuyf, Charles Pilette, Ghislaine Gayan-Ramirez, Bart Vanaudenaerde, Wim Janssens

**Affiliations:** 1 Lab of Respiratory Diseases, CHROMETA, KU Leuven, 3000 Leuven, Belgium; 2 Clinical and Experimental Endocrinology, CHROMETA, KU Leuven, 3000 Leuven, Belgium; 3 Institute of Experimental & Clinical Research—Pole of Pneumology, ENT and Dermatology, Université Catholique de Louvain (UCL), 1200 Brussels, Belgium

**Keywords:** vitamin D, COPD, PBEC, CSE

## Abstract

In chronic obstructive pulmonary disease (COPD), the bronchial epithelium is the first immune barrier that is triggered by cigarette smoke. Although vitamin D (vitD) has proven anti-inflammatory and antimicrobial effects in alveolar macrophages, little is known about the direct role of vitD on cigarette smoke-exposed bronchial epithelial cells. We examined the effects of vitD on a human bronchial epithelial cell line (16HBE) and on air–liquid culture of primary bronchial epithelial cells (PBEC) of COPD patients and controls exposed for 24 h to cigarette smoke extract (CSE). VitD decreased CSE-induced IL-8 secretion by 16HBE cells, but not by PBEC. VitD significantly increased the expression of the antimicrobial peptide cathelicidin in 16HBE and PBEC of both COPD subjects and controls. VitD did not affect epithelial to mesenchymal transition or epithelial MMP-9 expression and was not able to restore impaired wound healing by CSE in 16HBE cells. VitD increased the expression of its own catabolic enzyme CYP24A1 thereby maintaining its negative feedback. In conclusion, vitD supplementation may potentially reduce infectious exacerbations in COPD by the upregulation of cathelicidin in the bronchial epithelium.

## 1. Introduction

Cigarette smoking is the major risk factor for the development of chronic obstructive pulmonary disease (COPD) [1]. The bronchial epithelium is the first barrier encountered by harmful triggers such as cigarette smoke and infections. The epithelium operates as a physical and chemical barrier and may, by releasing reactive oxygen species and antimicrobial peptides, prevent particles and pathogens from invading the lung [2]. When pathogens invade the lung, a cellular response of neutrophils and macrophages is induced together with the production and release of cytokines and chemokines [3,4]. In addition, COPD is characterized by a decreased wound healing and a progressive airway remodeling in which matrix metalloproteases, especially MMP-9 play an important part [5,6]. Symptomatic COPD patients are mainly treated with inhaled bronchodilators (β-agonists, anticholinergics or a combination) and inhaled corticosteroids in case of repeated exacerbations [1]. These therapies have little impact on the bronchial epithelium as they mainly act on smooth muscle cells surrounding the airways and interact with immune cells, including eosinophils. As their potential in reducing the risk of exacerbations is rather limited, there is still a large need for more effective anti-inflammatory and antimicrobial treatments that could directly interact with the bronchial epithelium. 

Vitamin D (vitD), a known regulator of bone homeostasis, also exerts immunomodulatory effects [7,8]. Several in vitro studies showed anti-inflammatory and antimicrobial effects of vitD both on bronchial epithelial cells exposed to respiratory syncytial virus and in alveolar macrophages from smokers and nonsmokers [9,10]. In addition, Hansdottir et al. have shown that primary human tracheobronchial epithelial cells are able to locally produce and also inactivate 1,25(OH)_2_D via CYP27B1 and CYP24A1 respectively [11]. As the vitD receptor (VDR) is abundantly expressed in the bronchial epithelium [12], local vitD supplementation on the bronchial epithelium becomes an attractive target. So far, clinical studies using oral supplementation with vitD have shown an important reduction of exacerbation rate in severely vitD deficient patients [13]. The limiting factor in these studies might be the dose, as high doses of vitD increase the risk of hypercalcemia [14]. Local administration in the lung using aerosols with vitD or vitD analogues (like TX527), with less calcemic effects, could provide a new therapeutic option by directly interacting with the bronchial epithelium and reducing the risk of hypercalcemia. 

We therefore assessed the effects of cigarette smoke and vitD on inflammation, antimicrobial response, vitD metabolism and airway remodeling on a bronchial epithelial cell line. The effect of vitD and the vitD analogue TX527 on IL-8 secretion, expression of the antimicrobial peptide cathelicidin and vitD metabolism was tested in CSE-exposed primary bronchial epithelial cells (PBEC) from both unused donor lungs and end-stage COPD explant lungs. We found that vitD was able to upregulate cathelicidin in both cell-line and primary cell cultures, but downregulation of cigarette smoke extract (CSE)-induced IL-8 production was only seen in the 16HBE cell-line and not in PBEC. 

## 2. Materials and Methods

### 2.1. Cell Culture 

#### 2.1.1. The 16HBE Cell Line

The 16HBE14o^−^ (16HBE) cells were kindly provided by Dr. Gruenert (University of California, San Fran-cisco, CA, USA) and were cultured at 37 °C in 5% CO_2_ in DMEM/F12 medium (Life Technologies, Merelbeke, Belgium) supplemented with 5% fetal bovine serum (FBS), 100 U/mL Penicillin, 100 µg/mL Streptomycin, 2 mM L-glutamine and 2.5 µg/mL Amphotericin B. The 16HBE cells were used between passages 2–12. Medium was changed every 2–3 days and when confluent were split with 0.1% Trypsin-EDTA. Experiments were conducted using two independent experiments with three replicates each.

#### 2.1.2. Primary Bronchial Epithelial Cells

The use and collection of primary bronchial epithelial cells (PBEC) was approved by the Ethical Committee of University Hospital UZ Leuven (S51577/S55877) and written consent was given by all subjects. PBEC were isolated from the mainstem bronchus of unused donor lungs and lungs of COPD patients at the time of transplantation. A piece of bronchus was cleaned and incubated overnight in pronase (1 mg/mL Sigma-Aldrich, Hamburg, Germany) dissolved in RPMI 1640 medium, (Life Technologies). Afterwards, the tissue was transferred into a new tube with RPMI 1640 medium, shaken vigorously for 10 s and the tissue was removed from the tube. The resulting cells were centrifuged for 5 min at 350 g. The cell pellet was resuspended in 10 mL BEGM medium (Bronchial Epithelial Cell Growth medium Bulletkit, Lonza, Basel, Switzerland) supplemented with 100 U/mL Penicillin/Streptomycin, 2.5 µg/mL Amphotericin B, 100 nM retinoic acid (Sigma-Aldrich), 1.5 µg/mL BSA (Sigma-Aldrich) and singleQuots provided with the kit except for retinoic acid and gentamycin. Medium was changed every 2–3 days. Confluent cells were trypsinized and stored in 50%/50% BEGM/cryoprotective freezing medium (Lonza) in the vapors of liquid nitrogen. When needed, cells were taken out of storage, expanded and when confluent were seeded on collagen-coated (0.2 mg/mL) (Sigma-Aldrich) inserts, (Corning Transwell, Tewksbury, MA, USA) and cultured in Pneumacult Ex-Plus medium (StemCell Technologies, Vancouver, BC, Canada) according to the instructions with 100 U/mL Penicillin/Streptomycin, 2.5 µg/mL Amphotericin B for 6 days until confluent. Afterwards, cells were transferred to the air–liquid interface (ALI) for another 28 days to allow differentiation into a pseudostratified mucociliary airway epithelium. At this time, culture medium was changed to Pneumacult ALI-medium (StemCell Technologies) supplemented according to the instructions (ALI-supplement, hydrocortisone and heparin) and 100 U/mL Penicillin/Streptomycin and 2.5 µg/mL Amphotericin B. Cells were washed with DPBS without magnesium or calcium (Life Technologies) when needed (approximately 1×/week). Ciliary activity could be observed under the microscope for all used samples. Two examples of cultures are shown in Figure S1A.

### 2.2. Preparation Cigarette Smoke Extract 

Cigarette smoke extract (CSE) was made by bubbling smoke from 1 research cigarette with filter (3R4F research cigarettes, Kentucky Tobacco Research and Development Center, University of Kentucky, Lexington, KY, USA) through 10 mL complete DMEM/F12 with 3% FBS in case of 16HBE or in 10 mL Pneumacult ALI-medium medium in case of PBEC. To standardize CSE between experiments, the optical density (OD) was measured at 340 nm and CSE was adjusted (0.37 for cell line, 0.4 for PBEC, to obtain a similar increase in OD after CSE exposure). Afterwards, the CSE was filtered using a 0.2 mm pore filter (Millipore, Burlington, MA, USA). The obtained extract was considered to be 100% CSE and was further diluted in culture medium and used within 30 min.

### 2.3. CSE and Vitamin D Exposure

One day prior to exposure of 16HBE cells to CSE and/or vitamin D, medium for 16HBE cells was changed to DMEM/F12 with 3% FBS 100 U/mL Penicillin, 100 µg/mL Streptomycin, 2 mM L-glutamine and 2.5 µg/mL Amphotericin B. Culture conditions for PBEC were unchanged for exposure. Cells were exposed to vitD (either 1.25(OH)_2_D, 25(OH)D (Sigma-Aldrich) or the less calcemic vitD analogue TX527 (Prof. A.Verstuyf)) or vehicle (0.1% ethanol) for 30 min prior to 24 h exposure with 25% CSE with vitD or vehicle. PBEC were exposed via the basolateral compartment.

### 2.4. IL-8 ELISA

Cell supernatant was collected and stored until IL-8 concentration was measured using a sandwich IL-8 ELISA according to the instructions of the kit (Life Technologies). 

### 2.5. qPCR

Cell were washed with DPBS without calcium and magnesium (Life Technologies), scraped with a cell scraper and stored until RNA extraction in RLT buffer (Qiagen, Hilden, Germany) supplemented with β-mercaptoethanol (Bio-Rad, Hercules, CA, USA) for 16HBE cells and TRIzol (Life Technologies) for PBEC. RNA was isolated according to instruction of the RNeasy Mini kit (Qiagen, Germany). Afterwards, 1 µg RNA was reverse transcribed with Superscript III reverse transcriptase (Life Technologies). qPCR was performed using SYBR Green (Platinum^®^ SYBR^®^ Green qPCR SuperMix-UDG) (Life Technologies) with the Eco Real-Time PCR system (Illumina, Eindhoven, The Netherlands) and analyzed using the comparative cycle threshold method using GAPDH as a housekeeping gene. Primer sequences are listed in Table 1.

### 2.6. Wound Healing Assay

Fifty thousand 16HBE cells were seeded per well of the migration assay slides according to manufacturer’s instructions (IBIDI, Gräfelfing, Germany). Cells were grown until confluent, and 24 h before the assay cells were put into complete medium with 0.2% serum to stop proliferation. For the assay, the insert was removed and cells were again treated with CSE and vitD as described above. A picture was taken every hour. The gap between the two cell fronts was measured using ImageJ [15].

### 2.7. Statistics

Statistical analysis was performed with Graphpad Prism 7 (Graphpad, San Diego, CA, USA). To test the effect of CSE on IL-8 and the effect of vitD on CSE-induced IL-8 results were analyzed using the Mann–Whitney U test. This was also the case for the PBEC due to low sample size. For the rest of the analyses, a two-way ANOVA with a Tukey–Kramer post-hoc test for multiple group comparison was used. Data are shown as mean + SEM. For the PBEC, data are shown as median + IQR.

## 3. Results

### 3.1. Both 1,25(OH)2D and 25(OH)D Reduce CSE-Induced IL-8 by 16HBE Cells

**Figure 1:**
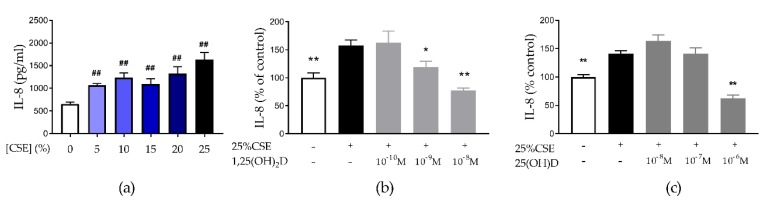
Effect of cigarette smoke extract (CSE) exposure and vitamin D (vitD) treatment on IL-8 secretion by 16HBE cells. (**a**) Increase in IL-8 secretion by 16HBE cells after 24 h CSE-exposure. (**b**,**c**) IL-8 secretion by 16HBE cells was decreased by 30 min pretreatment of vitD followed by 24 h exposure of CSE with 1,25(OH)_2_D and 25(OH)D respectively. ## *p* < 0.01 compared to 0%, * *p* < 0.05 compared to 25% CSE, ** *p* < 0.01 compared to 25% CSE. Data pertain to the result of two independent experiments with three replicates each.

CSE significantly increased IL-8 secretion by 16HBE cells in a dose-dependent manner (Figure 1a) without being toxic to the cells (Figure S2A. Treatment with 10^−9^ M and 10^−8^ M of 1,25(OH)_2_D was able to significantly reduce CSE-induced IL-8 secretion by 24.7% (*p* < 0.05) and 51% (*p* < 0.01), respectively (Figure 1b). Also, the precursor 25(OH)D significantly reduced CSE-induced IL-8 secretion at a dose of 10^−6^ M by 53.7% (*p* < 0.01) (Figure 1c). Both effective concentrations of 1,25(OH)_2_D and 25(OH)D were not toxic to the cells (Figure S2B,C). The vitD analogue TX527 also decreased CSE-induced IL-8 secretion, even at 10^−10^ M (Figure S3). We concluded that vitD exerts anti-inflammatory effects on 16HBE cells. Concentrations of 10^−8^ M 1,25(OH)_2_D and 10^−6^ M 25(OH)D were selected for further experiments. 

### 3.2. Both 1,25(OH)_2_D and 25(OH)D are Able to Induce Cathelicidin Expression in 16HBE Cells

**Figure 2:**
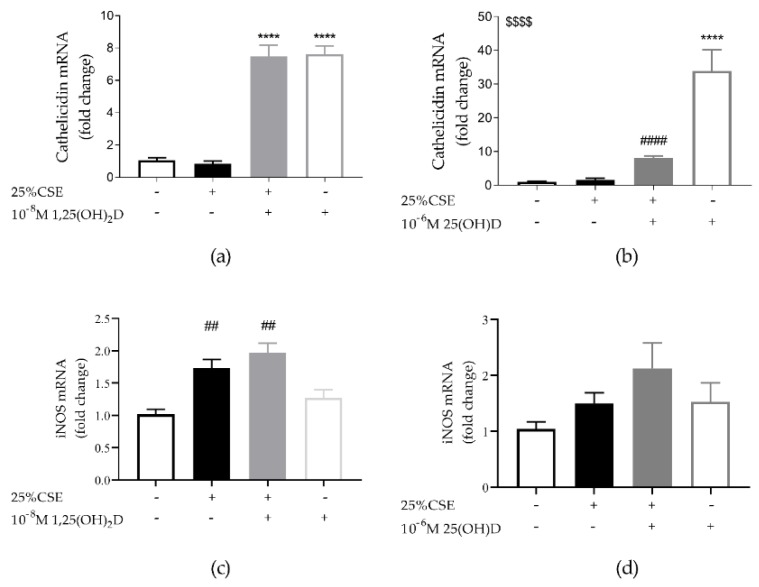
Effect of CSE and vitD exposure on mRNA expression of the antimicrobial peptide cathelicidin and inducible nitric oxide synthase (iNOS) in 16HBE cells. (**a**) Cathelicidin mRNA expression was upregulated by 1,25(OH)_2_D independently from 25% CSE. (**b**) 25(OH)D increased cathelicidin expression, but its expression was significantly reduced by CSE-exposure. (**c**) CSE significantly increased iNOS mRNA expression. There was a trend towards a 1,25(OH)_2_D effect as determined by a two-way ANOVA *p* = 0.06. (**d**) CSE and 25(OH)D had no significant effect on iNOS mRNA expression but there was a trend towards a CSE and 25(OH)D effect as determined by a two-way ANOVA (*p* = 0.08 and *p* = 0.09). **** *p* < 0.0001 effect of vitD, ## *p* < 0.01, #### *p* < 0.0001 effects of smoking. $$$$ interaction effect between 25(OH)D and CSE *p* < 0.01. Data pertain to the result of two independent experiments with three replicates each.

**Figure 3:**
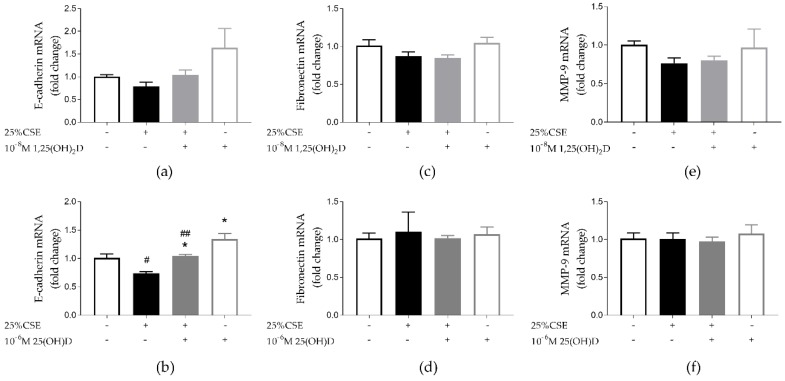
Effect of CSE and vitD on airway remodeling in 16HBE cells. (**a**) Neither 25% CSE, nor 1,25(OH)_2_D had a significant effect on E-cadherin mRNA expression; (**b**) 25% CSE significantly decreased E-cadherin expression. E-cadherin mRNA expression was significantly upregulated by 25(OH)D. (**c**,**d**) Neither 25% CSE, nor 1,25(OH)_2_D or 25(OH)D had any effect on fibronectin mRNA expression. (**e**,**f**) Neither 25% CSE, nor 1,25(OH)_2_D or 25(OH)D had any effect on MMP-9 mRNA expression. * *p* < 0.05 effect of vitD, # *p* < 0.05, ## *p* < 0.01 effect of 25% CSE. Data pertain to the result of two independent experiments with three replicates each.

It was found that 1,25(OH)_2_D significantly upregulated cathelicidin mRNA expression in 16HBE cells both when cells were exposed to 1,25(OH)_2_D alone (*p* < 0.0001) or in combination with CSE (*p* < 0.0001) (Figure 2). Also, 25(OH)D significantly upregulated cathelicidin mRNA expression compared to controls (*p* < 0.0001). However, when cells were exposed to both 25(OH)D and CSE, cathelicidin expression was significantly reduced compared to exposure to 25(OH)D alone (*p* < 0.001). In addition, we also assessed inducible nitric oxide synthase (iNOS) mRNA expression as NO has antimicrobial properties and iNOS is known to be vitD sensitive. CSE upregulated iNOS mRNA expression (*p* < 0.01 for Figure 3c; *p* = 0.08 for Figure 3d), while a trend towards an upregulation of iNOS mRNA was observed after vitD (Figure 3c,d, *p* = 0.06 and *p* = 0.09, respectively). We concluded that vitD has antimicrobial potential on 16HBE cells via the upregulation of cathelicidin and possibly via iNOS.

### 3.3. Effect of CSE and Vitamin D on Airway Remodeling and Wound Healing in 16HBE Cells

**Figure 4:**
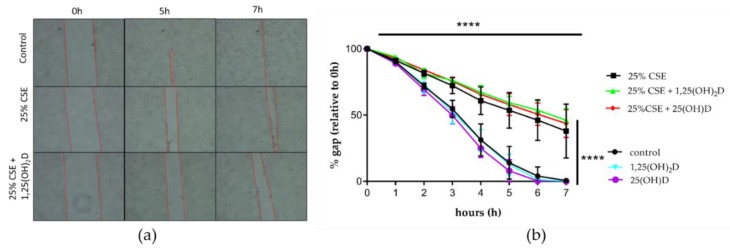
Effect of CSE and VitD on wound healing in 16HBE cells. (**a**) Illustration of migration assay. (**b**) CSE significantly reduced wound healing capacity of 16HBE cells. Neither 1,25(OH)_2_D, nor 25(OH)D had an effect on wound healing. Data are the result of three independent experiments.

Epithelial to mesenchymal transition (EMT) is characterized by a loss of epithelial markers (like E-cadherin) and an increase in mesenchymal markers (like fibronectin). Exposure to 25(OH)D significantly upregulated E-cadherin mRNA expression (*p* < 0.05) (Figure 3b). CSE significantly decreased E-cadherin mRNA expression independent of 25(OH)D treatment (*p* < 0.05 and *p* < 0.01) (Figure 3b). Similar trends were visible for 1,25(OH)_2_D, however, these were not statically significant (Figure 3a Neither vitD, nor CSE exposure had any effect on fibronectin mRNA expression (Figure 3c,d)). Protein levels of E-cadherin and Fibronectin for experiments with 1.25(OH)_2_D can be found in Figure S4. Likewise neither CSE, nor vitD exposure affected MMP-9 mRNA expression (Figure 3e,f). Finally, we tested the functional effect of vitD in a wound healing assay. It was found that 25% CSE significantly decreased the wound healing capacity of 16HBE cells, but 1,25(OH)_2_D or 25(OH)D were not able to restore migration capacity (Figure 4a,b). We concluded that vitD has little effect on airway remodeling as addressed by a wound healing assay.

### 3.4. Effect of CSE and Vitamin D on Vitamin D Metabolism

**Figure 5:**
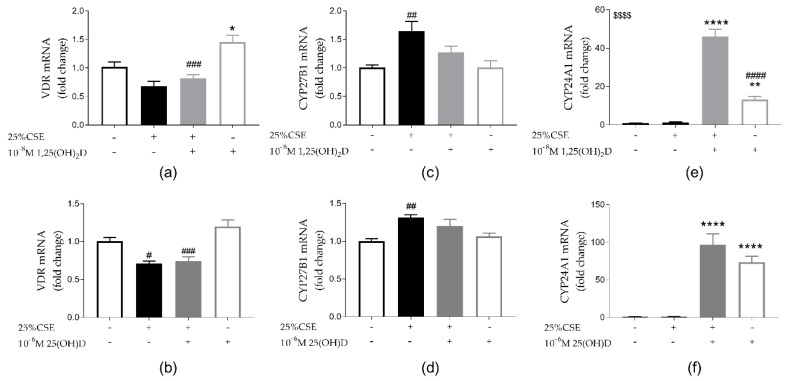
Effect of CSE and vitD on vitD metabolism in 16HBE cells. Both 1,25(OH)_2_D (**a**) and 25(OH)D (**b**) upregulate VDR mRNA expression (*p* < 0.05 and *p* = 0.13 respectively). CSE exposure reduces VDR mRNA expression independently of vitD exposure but this was only significant with 25(OH)D (5b, *p* < 0.05) while a trend was observed for 1.25(OH)D2 (5a, *p* = 0.073). (**c**,**d**) CYP27B1 mRNA expression was significantly upregulated by CSE exposure. (**e**,**f**) CYP24A1 mRNA expression is significantly upregulated by both 1,25(OH)_2_D and 25(OH)D. In case of 1,25(OH)_2_D, CYP24A1 mRNA expression was even further upregulated by the combination of CSE and 1,25(OH)_2_D exposure. * *p* < 0.05, ** *p* < 0.01, **** *p* < 0.0001 effect of vitD, # *p* < 0.05, ## *p* < 0.01, ### *p* < 0.001 effect of 25% CSE. $$$$ interaction effect for CYP24A1 between 25% CSE and 1,25(OH)_2_D *p* < 0.0001. Data pertain to the result of two independent experiments with three replicates each.

VDR was significantly upregulated by exposure to 1,25(OH)_2_D (*p* < 0.01) and a trend towards VDR mRNA upregulation was seen upon treatment with 25(OH)D (*p* = 0.13) (Figure 5a,b). CSE exposure reduced VDR mRNA expression independent of vitD. In addition, CSE inhibited the upregulation of VDR by 1,25(OH)_2_D (*p* < 0.001). Besides downregulation of mRNA VDR expression, CSE exposure also induced CYP27B1, the activator of vitD (Figure 5a–d). Exposure to vitD alone (both 1,25(OH)_2_D and 25(OH)D) or vitD in combination with CSE significantly upregulated CYP24A1, inactivator of vitD, expression (Figure 3e,f). For 1,25(OH)_2_D, the combination of vitD and CSE exposure significantly further upregulated CYP24A1 expression compared to 1,25(OH)_2_D alone (*p* < 0.0001) (Figure 5e). However, this was not the case for 25(OH)D. Even though the potential of vitD might be increased by the upregulation of CYP27B1 by CSE, VDR was downregulated by CSE and CYP24A1 was upregulated by vitD thereby possibly limiting the effects of vitD on 16HBE cells.

### 3.5. Effect of CSE and Vitamin D on PBEC from Unused Donor Lungs and COPD Explant Lungs

PBECs were used from both unused donor lungs and COPD explant lungs. Patients were matched for age and gender (Table 2). Unused donor lungs rejected for emphysema were excluded. Reasons for not using these donor lungs were infection, logistics and a recipient that died before implementation. The fourth piece of donor tissue was a piece of excessive donor bronchus that was not needed due to extensive trimming before implementation. All COPD patients had a severely impaired lung function as measured by FEV_1_%, FVC% FEV_1_%/FVC% and DLCO%. Three COPD patients already received oral vitamin D supplementation before lung transplantation. (Table 2).

**Figure 6:**
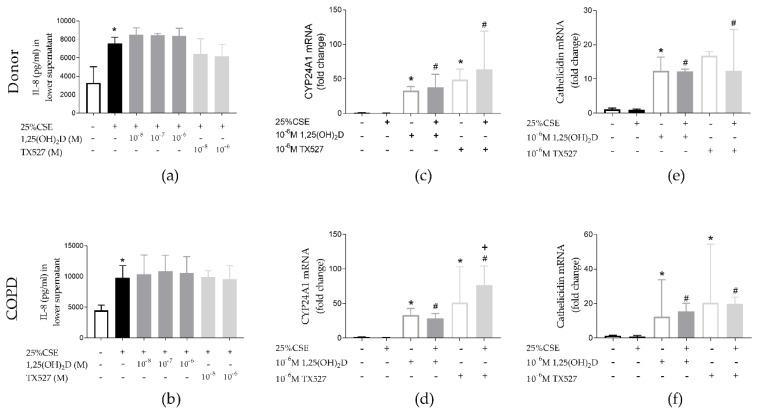
Effect of CSE and vitD exposure on primary bronchial epithelial cells (PBEC) of unused donor lungs (upper panels) and COPD explant lungs (lower panels). CSE significantly increased IL-8 secretion by PBEC of both controls (**a**) and COPD patients (**b**). Neither 1,25(OH)_2_D nor TX527 reduced CSE-induced IL-8 secretion. Both 1,25(OH)_2_D and TX527 significantly upregulated mRNA expression of CYP24A1 (**c**,**d**) and cathelicidin (**e**,**f**) in PBEC from unused donor lungs and COPD patients. Concentrations of all drugs on the graph are 10^−6^ M; *n* = 4 per group. * *p* < 0.05 compared to control, # *p* < 0.05 compared to 25% CSE, + *p* < 0.05 compared to 1.25(OH)_2_D.

25% CSE increased IL-8 secretion by PBEC both from unused donor lungs and COPD explant lungs (*p* < 0.05). Neither 1,25(OH)_2_D, nor the less calcemic vitD analogue TX527 were able to significantly decrease CSE-induced IL-8 secretion. (Figure 6a,b). Both 1,25(OH)_2_D and TX527 upregulated CYP24A1 (*p* < 0.05) (Figure 6c,d) and cathelicidin mRNA expression (*p* < 0.05) (Figure 6e,f) in PBEC from both unused donor lungs and COPD explant lungs. No significant differences were observed in VDR, CYP27B1 and iNOS mRNA expression (Figure S5). Even though the anti-inflammatory effects of vitD could not be confirmed in PBEC, the antimicrobial potential was maintained.

## 4. Discussion

We demonstrate the anti-microbial potential of vitamin D (1,25(OH)_2_D and 25(OH)D) and of the vitD analogue TX527 on airway epithelial cells by the upregulation of the antimicrobial peptide cathelicidin. The anti-inflammatory effect of vitD and vitD analogue, TX527, on the bronchial epithelial cell line 16HBE, could not be confirmed on the PBEC from unused donor lungs and COPD explant lungs. Both 1,25(OH)_2_D and TX527 also upregulated CYP24A1 expression indicating that 1,25(OH)_2_D and TX527 were active on PBEC but perhaps insufficient to have an effect on the inflammatory pathway. 

The bronchial epithelium is a key player in the pathogenesis of COPD as it serves as a first barrier against harmful triggers and acts as an initiator of inflammatory processes. COPD is characterized by a predominantly neutrophilic inflammation with IL-8 serving as one of the main neutrophil attractants [16,17]. Several studies, including the present study, have shown that bronchial epithelial cells (cell lines, PBEC and primary human small airway epithelial cells) secrete IL-8 upon exposure to CSE [18,19,20,21]. An anti-inflammatory effect of vitD is described by our own group on CSE-exposed THP-1 macrophages and alveolar macrophages from smokers and non-smokers [10], by Hansdottir et al. on PBEC in response to respiratory syncytial virus and by Mulligan et al. in response to CSE in human nasal epithelial cells [9,22]. Moreover, intratracheal instillation of vitD together with LPS inhibited inflammatory cell recruitment into the broncho-alveolar lavage fluid in a hamster model of acute lung injury. The latter results suggest that direct interaction of vitD with lung epithelial cells can also decrease inflammation in vivo [23]. Despite the anti-inflammatory effect of vitD on CSE-exposed 16HBE cells, we could not confirm these results on PBEC from unused donor lungs or COPD explant lungs. This indicates that the type of cells and the nature of the trigger are important experimental determinants that one has to consider before making general conclusions. 

CSE has not only been shown to increase inflammation, but also to impair bacterial defense. We previously showed that phagocytosis was impaired in alveolar macrophages of smokers compared to non-smokers [10]. As respiratory infections play an important role in the development and progression of COPD, the increase in antimicrobial peptides (AMP) could improve bacterial clearance and might help in preventing exacerbations or reduce exacerbation severity. The bronchial epithelium is able to release AMPs [24]. In COPD epithelial cultures, the upregulation of AMPs in response to nontypeable *Haemophilus influenzae* (NTHi) is decreased, while CSE decreases NTHi-induced AMP expression in both COPD and non-COPD epithelial cultures [25]. In the present study, we could show that both in cell lines and PBEC (from both unused donor lungs and COPD explant lungs), vitD and the vitD analogue TX527 are able to upregulate expression of the antimicrobial peptide cathelicidin, independent of CSE exposure. Cathelicidin has previously been shown to be upregulated by vitD [11,26] and is active against both viruses and bacteria including Rhinoviruses and *Mycobacterium* tuberculosis [27,28]. As NO is another anti-microbial agent, we also assessed mRNA levels of iNOS, known to be vitamin D sensitive [29]. While iNOS was upregulated by CSE, a trend toward upregulation by vitD was detected in 16HBE cells. These results suggest that vitD, even in a smoke-exposed environment, has the potential to produce antimicrobial peptides and other antimicrobial agents like NO via iNOS, and thereby possibly limit infections and slow down the progression of COPD. Several randomized control trials have investigated the effect of vitD supplementation in COPD patients. A recent meta-analysis showed that vitD supplementation reduced the exacerbation rate in severely vitD deficient COPD patients (serum 25(OH)D < 10 ng/mL), while no beneficial effects were found on lung function [13]. The reduction in exacerbation rate might be explained by the upregulation of AMPs like cathelicidin and possibly of NO via iNOS. One may even speculate that this upregulation is relatively more important in COPD given the reduced phagocytic capacity of macrophages and neutrophils with smoke exposure. These results seem contradictory to our recent findings showing that infection with NTHi was more rapidly cleared in vitD deficient mice compared to vitD sufficient mice. However, it is important to acknowledge that several vitD responsive genes in humans like cathelicidin are not vitD responsive in mice e.g., cathelecidin-related antimicrobial pepetide (CRAMP). This discrepancy between humans and mice is likely to reflect the limitation of using animal models to mimic human situations [30]. 

Another hallmark of COPD is airway remodeling and delayed wound repair. COPD is characterized by small airways remodeling, increased mucus production, increased permeability, changed cell composition and sub-epithelial changes such as airway smooth muscle cell hyperplasia and peribronchial fibrosis [31,32,33,34]. In this study, we investigated if CSE caused epithelial to mesenchymal transition (EMT) and increased MMP-9 expression and whether vitD could convert the effects. In our experiments, CSE did decrease E-cadherin mRNA levels, an epithelial marker, but we found no upregulation of fibronectin mRNA levels, a mesenchymal marker. This is in contrast with the studies of Eurlings et al. and Milara et al. who did describe EMT after CSE exposure in lung epithelial cells [35,36]. However, in these studies cells were exposed to CSE for 48–72 h whereas in our study, 24 h exposure is likely too short to see effects on mesenchymal markers. In contrast to what we expected, MMP-9 expression, a powerful metalloproteinase, was not upregulated by CSE nor altered by vitD exposure. MMP-9 expression has previously been shown to be increased in alveolar macrophages of COPD patients exposed to cigarette smoke [37]. We also explored functional remodeling by wound healing, which is known to be impaired in COPD [38,39]. We confirmed a significant reduction in wound healing capacity with CSE, but even though vitD is known to associate with improved wound healing, we could not demonstrate any beneficial effect of vitD [40]. These findings do corroborate with another study showing decreased would healing capacity of human bronchial epithelial cells when exposed to TGF-β and calcitriol [41].

The duration of anti-inflammatory or antimicrobial effects of vitD (1,25(OH)_2_D, 25(OH)D as well as TX527) seems to be limited by an increased expression of the catabolic enzyme CYP24A1. Moreover, the potential of vitD to exert beneficial effects may be further reduced by an impaired upregulation of VDR in 16HBE by 1,25(OH)_2_D and 25(OH)D, when exposed to CSE. While no significant differences were found on PBEC, PBEC from COPD patients tend to show a reduced VDR mRNA level compared to PBEC from unused donor lungs (*p* = 0.0571). Our results are consistent with those of Ishii et al. who described a downregulation of VDR by CSE in A549 cells, but contrast with the results of Liu et al. who showed that TLR-triggering of TLR1/2 upregulated VDR expression [42,43]. On the other hand, CSE was found to upregulate CYP27B1 expression in 16HBE cells, facilitating the conversion of 25(OH)D to the active form 1,25(OH)_2_D. No significant changes were observed in PBEC. Buonfiglio et al. and Mulligan et al. showed a downregulation of CYP27B1 in PBEC and sinonasal epithelial cells, respectively, after CSE exposure [22,44]. Overall, one may conclude that the upregulation of the catabolic CYP24A1 and the downregulation of VDR are potentially limiting the effects of vitD. As this hypothesis is made on mRNA expression only, protein expression and enzyme activity are needed to draw more formal conclusions. 

Although 16HBE cells are frequently used to model the bronchial epithelium, they do lack key features of human bronchial epithelial cells. Although they do express tight junction proteins and have a cobblestone appearance, differentiation into a pseudostratified epithelium is controversial [45,46,47,48]. Because of these limitations of 16HBE cells, we also collected PBEC from unused/declined donor lungs as well as from COPD patients. Although smoking history was not known for all unused donor lungs, better knowledge of patient characteristics (specifically of the COPD patient cells) and the ability of cells to differentiate into all relevant cell types of the bronchial epithelium, make the use of PBEC more advantageous. Not all effects of vitD on the 16HBE cells could be confirmed on primary cell cultures. One logical explanation is that all cell lines change their characteristics over time. In our study however, we were also limited by the experimental conditions. In particular, the addition of fluid in the apical compartment after ALI caused too much stress for the cells and impaired ciliary function. We therefore exposed our PBEC with CSE and vitD via the basal compartment and for a maximal period of 24 h, which may have caused our negative observations. A smoking chamber and vitamin D aerosols are likely the optimal setting to replicate our experiments in the future. Another explanation could be that the patients from which cells were used were vitamin D insufficient while a clinical effect is only observed in severely vitamin D deficient patients [13]. In our center, vitamin D deficient COPD patients are routinely supplemented with vitamin D because of the clinical benefit. Therefore, the number of vitamin D deficient COPD patients is limited. However, the increase in cathelicidin with vitD supports beneficial effects in this population, an effect that should not be neglected.

## 5. Conclusions

In this study, we explored the anti-inflammatory and antibacterial potential of vitD on bronchial epithelial cells in a cigarette smoke environment. Our data illustrate that vitD may interact with the inflammatory process initiated by the bronchial epithelium, particularly through its effects on antimicrobial peptides. 

## Figures and Tables

**Table 1:** Primer list.

Gene	Forward Primer	Reverse Primer
GAPDH	TGGTATCGTGGAAGGACTCA	CCAGTAGAGGCAGGGATGAT
VDR	GATTGGAGAAGCTGGACGAG	GTTCGTGTGAATGATGGTGGA
CYP27B1	CGCACTGTCCCAAAGCTG	CGGAGCTTGGCAGACATC
CYP24A1	GTGACCATCATCCTCCCAAA	AGTATCTGCCTCGTGTTGTATG
Cathelicidin	GGGCTCCTTTGACATCAGTT	AGCAGGGCAAATCTCTTGTT
iNOS	TTCAGTATCACAACCTCAGCAAG	TGGACCTGCAAGTTAAAATCCC
Fibronectin	AAACCAATTCTTGGAGGAGG	CCATAAAGGGCAACCAAGAG
E-cadherin	GAAGGTGACAGAGCCTCTGGAT	GATCGGTTACCGTGATCAAAATC
MMP-9	GCACGACGTCTTCCAGTACC	CAGGATGTCATAGGTCACGTAGC

Abbreviations: GAPDH: Glyceraldehyde 3-phosphate dehydrogenase VDR: vitamin D receptor; CYP27B1: 1-alpha-hydroxylase; CYP24A1: 24-hydroxylase; iNOS: inducible nitric oxide synthase MMP-9: Matrix Metalloproteinase-9.

**Table 2:** Patient characteristics.

	Donor	COPD	*p*-Value
Age (years)	57 (55–58)	57 (53–60)	0.873
gender (M/F)	1/3	1/3	>0.999
FEV_1_%	NA	24.5 (21.0–25.8)	NA
FEV_1_%/FVC%	NA	34 (24.75–30.25)	NA
FVC%	NA	62.5 (56.5–64.0)	NA
DLco%	NA	36 (27–39)	NA
Smoking history (Y/N/U)	1/2/1	4/0/0	
VitD supplementation (Y/N/U)	0/0/4	3/1/0	NA
Serum 25(OH)D pretransplant µg/L	NA	27 (21–43)	NA

Abbreviations: M = male, F = female, Y = yes, N = no, U = unknown, NA = not applicable, FEV_1_% = % predicted forced expiratory volume in 1 s, FVC% = % predicted forced vital capacity, FEV_1_%/FVC% = Tiffeneau index, DLco% = % predicted diffusion capacity DLco% and pretransplant serum 25(OH)D were unknown for one chronic obstructive pulmonary disease (COPD) patient. Data are shown as median (IQR).

## Supplementary Materials

The following are available online at https://www.mdpi.com/2072-6643/11/9/2138/s1, Figure S1: Examples of differentiated PBEC cultures from an unused donor lung (**A**) and a COPD explant lung (**B**) Cilia are indicated by the blue arrow (40× magnification).; Figure S2: Cytotoxicity test of CSE and vitamin D exposure on 16HBE; Figure S3: Effect of CSE and TX527 exposure on 16HBE; Figure S4: Effect of CSE and 1.25(OH) on E-cadherin and Fibronectin protein levels in 16HBE cells; Figure S5: Effect of CSE and 1.25(OH)2D /TX527 exposure on VDR, CYP27B1 and iNOS expression in PBEC of unused donor lungs (upper panels) and COPD explant lungs (lower panels).
