# Supplementary material for: Vitamin D Modulates the Response of Bronchial Epithelial Cells Exposed to Cigarette Smoke Extract
Source: Nutrients. 2019 Sep 6;11(9):2138. doi: 10.3390/nu11092138 (PMC6770037; doi:10.3390/nu11092138)
Supplement: Supplementary file 1 [file nutrients-11-02138-s001.zip › nutrients-565763 supplementary/Supplementary File 1.docx]

S1: Differentiation of PBEC


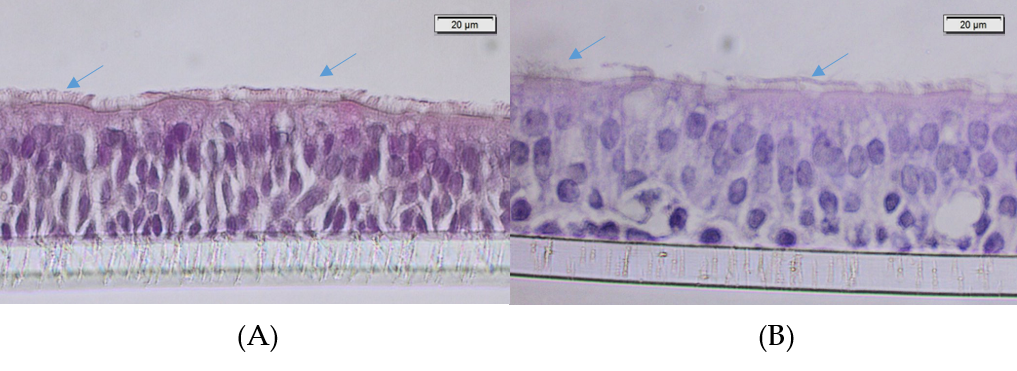


**Supplementary Figure S1**. Examples of differentiated PBEC cultures from an unused donor lung (**A**) and a COPD explant lung (**B**) Cilia are indicated by the blue arrow (40× magnification).
