# Supplementary material for: Vitamin D Modulates the Response of Bronchial Epithelial Cells Exposed to Cigarette Smoke Extract
Source: Nutrients. 2019 Sep 6;11(9):2138. doi: 10.3390/nu11092138 (PMC6770037; doi:10.3390/nu11092138)
Supplement: Supplementary file 1 [file nutrients-11-02138-s001.zip › nutrients-565763 supplementary/Supplementary File 2.docx]

## **S2: Cytotoxicity tests 16HBE cells**

16HBE cells were seeded in a 96-well plate. Upon at least 80% confluence, cells were put at 3% serum DMEM/F12 medium for 24 h. Afterwards, cells were exposed to 25% CSE for 24 h. In the experiments with vitamin D, cells were first exposed to vehicle or vitamin D (either 1.25(OH)_2_D , 25(OH)D or TX527) before being exposed for 24 h to CSE with or without vitamin D. After exposure, cells were washed with DPBS without calcium and magnesium. Afterwards, WST-1 (diluted 1/20)(Roche Diagnostics, Belgium) was added to the cells. After 1 hour, cell supernatant was taken and measured at 450 nm using a spectrophotometer (Bio-Rad Model 680XR microplate reader). Control cells (without CSE or vitamin D) were considered to be 100%. Conditions were considered to be toxic when viability was < 80%.


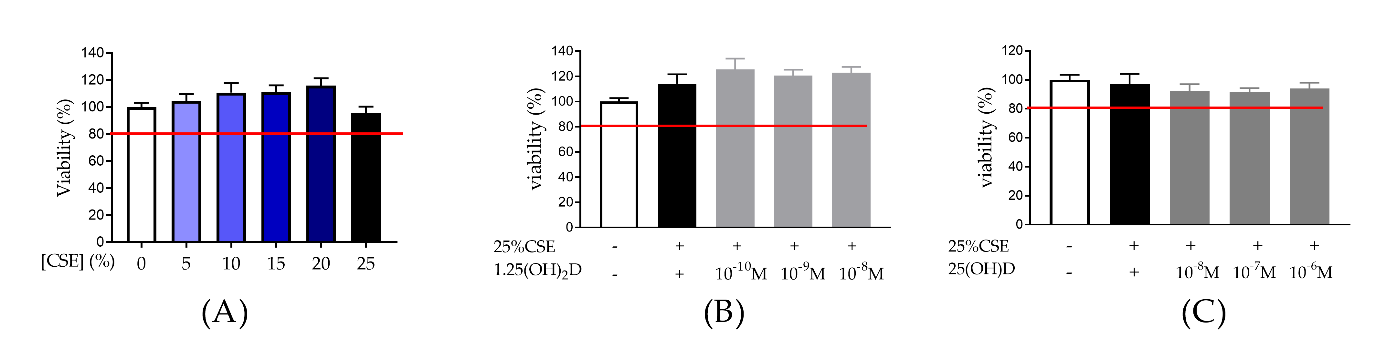


**Supplementary Figure S2**. Cytotoxicity test of CSE and vitamin D exposure on 16HBE. **A**. WST-1 for different CSE concentrations showed no toxicity. **B**. The used concentrations of 1.25(OH)_2_D showed no cytotoxicity. **C**. The used concentrations of 25(OH)D showed no cytotoxicity N = 6
