# Supplementary material for: Vitamin D Modulates the Response of Bronchial Epithelial Cells Exposed to Cigarette Smoke Extract
Source: Nutrients. 2019 Sep 6;11(9):2138. doi: 10.3390/nu11092138 (PMC6770037; doi:10.3390/nu11092138)
Supplement: Supplementary file 1 [file nutrients-11-02138-s001.zip › nutrients-565763 supplementary/Supplementary File 3.docx]

## **S3: CSE and TX527 exposure**

One day prior to exposure of 16HBE cells to CSE and/or TX527, medium for 16HBE cells was changed to DMEM/F12 with 3% FBS 100 U/mL Penicillin, 100 µg/mL Streptomycin, 2mM L-glutamine and 2.5 µg/mL Amphotericin B. Cells were exposed to TX527 or vehicle (0.1% ethanol) for 30 min prior to 24 h exposure with 25% CSE with TX527 or vehicle.


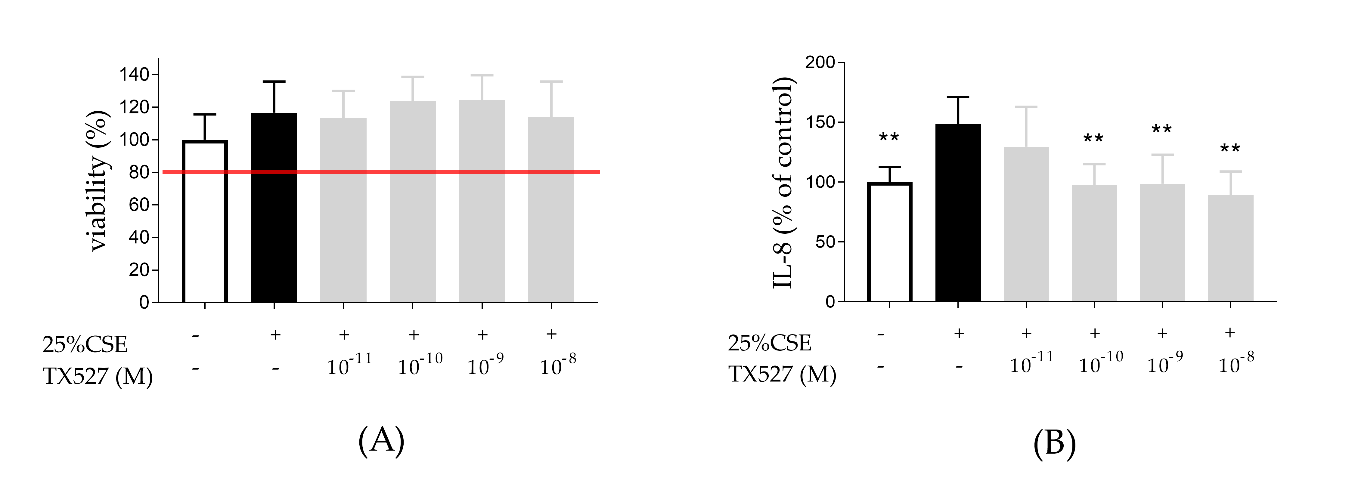


**Supplementary Figure S3**. Effect of CSE and TX527 exposure on 16HBE. **A**. WST-1 for different TX527 concentrations showed no toxicity. **B**. TX527 significantly reduced CSE-induced IL-8 secretion by 16HEB cells ** *p* < 0.05 compared to 25%CSE only N = 6
