# Supplementary material for: Vitamin D Modulates the Response of Bronchial Epithelial Cells Exposed to Cigarette Smoke Extract
Source: Nutrients. 2019 Sep 6;11(9):2138. doi: 10.3390/nu11092138 (PMC6770037; doi:10.3390/nu11092138)
Supplement: Supplementary file 1 [file nutrients-11-02138-s001.zip › nutrients-565763 supplementary/Supplementary File 4.docx]

## **S4: E-cadherin and Fibronectin protein levels**

Material and methods

Western Blot

Cells were lysed in Cell extraction buffer (Life Technologies) supplemented with 1 tablet/ 10mL cOmplete™, Mini Protease Inhibitor Cocktail (Sigma-Aldrich) and 1 tablet/ 10 mL PhosSTOP (Roche). Afterwards cells were centrifuged at 1,2000g for 15min at 4 °C. Supernatant was transferred to a new tube and stored at −80 °C. Samples were loaded on a 8% BIS-TRIS Plus gel (Invitrogen) for 45min at 165V. Afterwards the proteins were transferred using the iBlot 2 gel transfer device (Thermofisher Scientific) using the iBlot™ 2 Transfer Stacks, PVDF (Invitrogen). Transfer was done as follows using P0 (20-25V for 7 minutes). Afterwards, membranes were blocked in 5% NFDM-TBST for 1h after which the membrane was incubated overnight at 4 °C with the primary antibody. Anti- E-cadherin (BD Bioscience) was diluted 1/4000, anti-fibronectin (Abcam) was diluted 1/1000 and anti-GAPDH (cell signaling) was diluted 1/1000. The following day, membranes were washed 3 times with TBST for 5 min after which the membrane was incubated with secondary antibody 1/1000 (DAKO) for 1h at room temperature. Afterwards, membranes were again washed 3 times with TBST for 5min and once with TBS for 5min. Finally, blots were visualized using ECL^™^ Prime Western Blotting Detection Reagent (GE Healthcare).

**
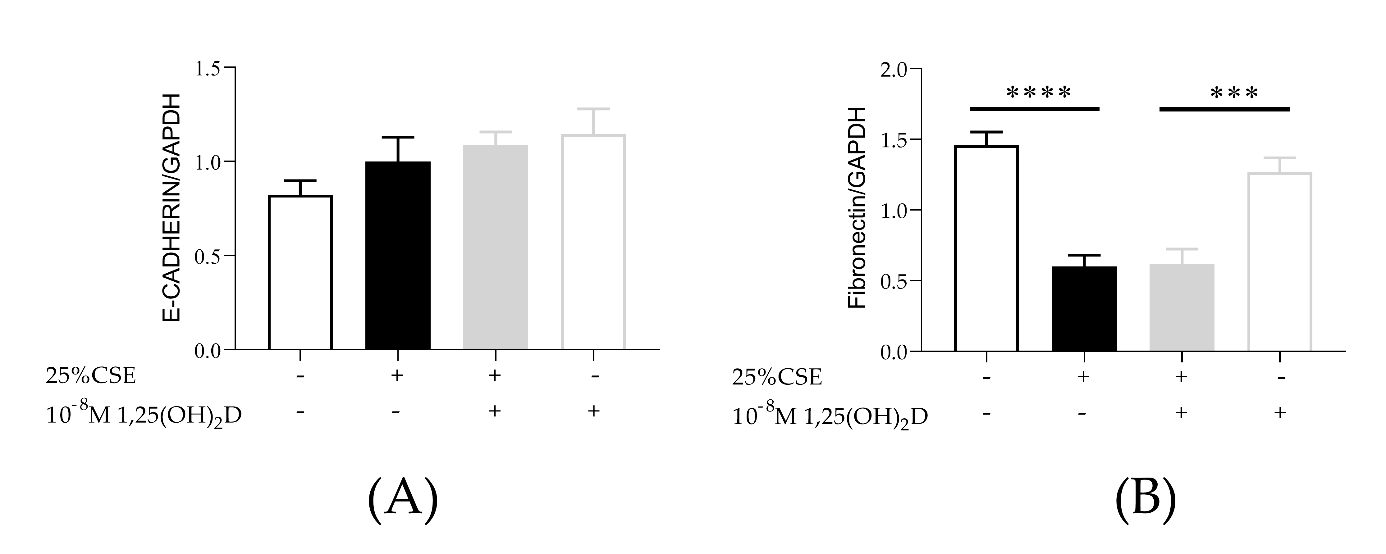
**

**Supplementary Figure S4**. Effect of CSE and 1.25(OH) on E-cadherin and Fibronectin protein levels in 16HBE cells. (**A**) Protein levels of E-cadherin were not significantly altered by CSE or 1.25(OH)2D, although there was a non-significant trend towards a vitamin D effect as determined by a Two-Way ANOVA *p* = 0.0686. (**B**) Protein levels of fibronectin were significantly decreased by CSE exposure independent of 1.25(OH)2D **** *p* < 0.0001 *** *p* < 0.001 Data pertain to the result of 2 independent experiments with 3 replicates each
