# Supplementary material for: Vitamin D Modulates the Response of Bronchial Epithelial Cells Exposed to Cigarette Smoke Extract
Source: Nutrients. 2019 Sep 6;11(9):2138. doi: 10.3390/nu11092138 (PMC6770037; doi:10.3390/nu11092138)
Supplement: Supplementary file 1 [file nutrients-11-02138-s001.zip › nutrients-565763 supplementary/Supplementary File 5.docx]

## **S5: VDR, CYP27B1 and iNOS mRNA expression in PBEC**

**
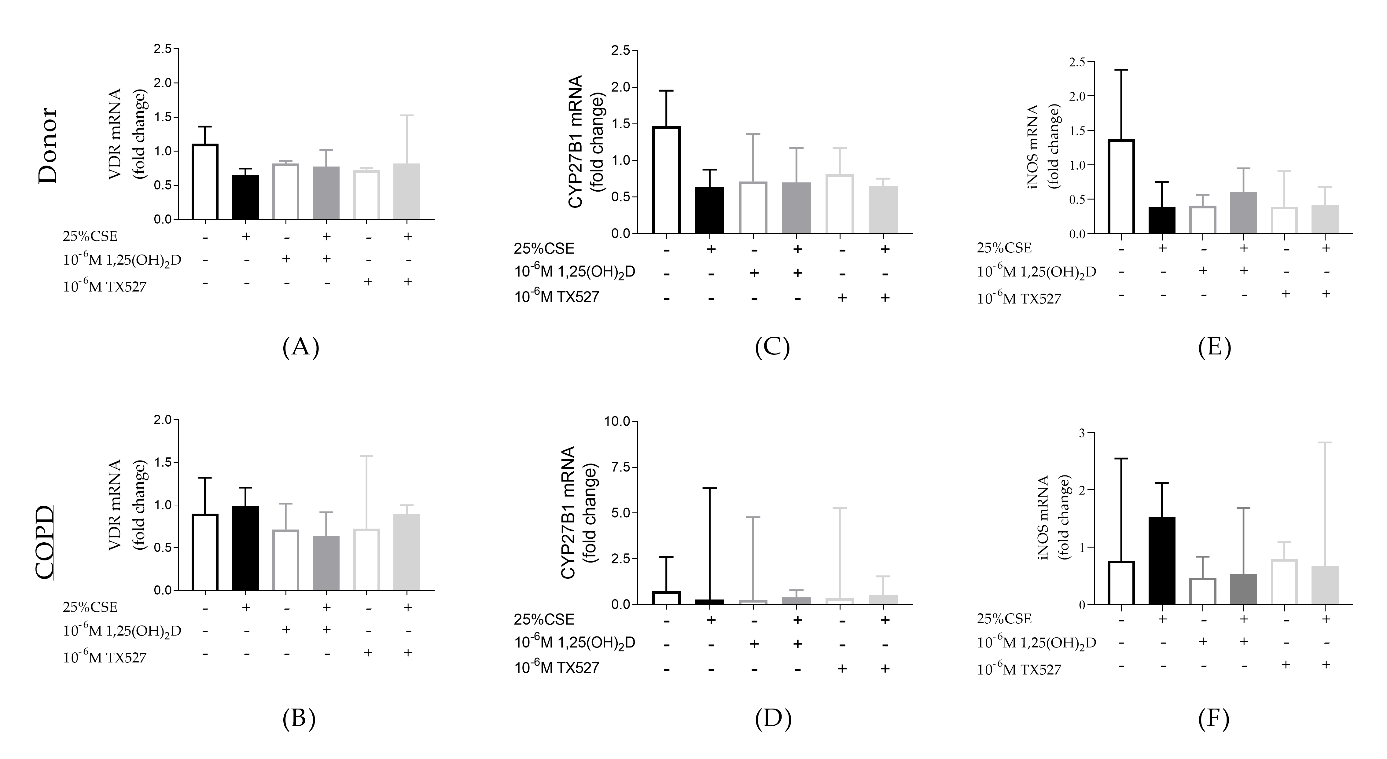
**

**Supplementary Figure S5.** Effect of CSE and 1.25(OH)_2_D /TX527 exposure on VDR, CYP27B1 and iNOS expression in PBEC of unused donor lungs (upper panels) and COPD explant lungs (lower panels). **A**. There was no significant effect of CSE, 1.25(OH)_2_D or TX527 on VDR mRNA expression levels in PBEC from donors. **B**. There was no significant effect of CSE, 1.25(OH)_2_D or TX527 on VDR mRNA expression levels in PBEC from COPD explant lungs. **C**. There was no significant effect of CSE, 1.25(OH)_2_D or TX527 on CYP27B1 mRNA expression levels in PBEC from donors. **D**. There was no significant effect of CSE, 1.25(OH)_2_D or TX527 on VDR mRNA expression levels in PBEC from COPD explant lungs. **E**. There was no significant effect of CSE, 1.25(OH)_2_D or TX527 on iNOS mRNA expression levels in PBEC from donors. **F**. There was no significant effect of CSE, 1.25(OH)_2_D or TX527 on iNOS mRNA expression levels in PBEC from COPD explant lungs. N = 4 per group
